# [1,4]Ditellurino[2,3-*b*:5,6-*b*′]di­pyrazine

**DOI:** 10.1107/S2414314622006228

**Published:** 2022-06-24

**Authors:** Donna Franklin, Aundrea Lee, Frank R. Fronczek, Thomas Junk

**Affiliations:** a Department of Chemistry, Lafayette, LA 70403, USA; b Department of Chemistry, Louisiana State University, Baton Rouge, LA 70803, USA; University of Aberdeen, Scotland

**Keywords:** tellurium, pyrazine, heterocyclic, supra­molecular, crystal structure

## Abstract

[1,4]Ditellurino[2,3-*b*:5,6-*b*′]di­pyrazine represents the first reported [1,4]chalcogena[2,3-*b*:5,6-*b*′]di­pyrazine containing a heavy chalcogens The asymmetric unit consists of three mol­ecules. In contrast to its sulfur analog, which is planar [Lynch *et al.* (1994 ▸) *Cryst. Struct. Commun.* **50**,1470–1472], C_8_H_4_N_4_Te_2_ is folded along the Te⋯Te axis to accommodate the larger chalcogenide atoms. The dihedral angle between the two Te_2_C_2_ rings of the central ring is 57.9° (mean of three). C—Te bond lengths range from 2.1105 (16) Å to 2.1381 (17) Å, in good agreement with those predicted by their covalent radii. All Te atoms are involved in inter­molecular Te⋯N contacts, with distances in the range 2.894 (2) to 2.963 (2) Å. These result in a spiral supra­molecular assembly, forming helical columns.

## Abstract 2

The title compound, C_8_H_4_N_4_Te_2_, is the first reported [1,4]tellura[2,3-*b*:5,6-*b*′]di­pyrazine. Three independent unit-cell mol­ecules are folded along their Te⋯Te axes, with an average angle φ = 57.9°. C—Te—C angles range from 91.48 (6) to 93.80 (6)°. Inter­molecular N⋯Te bonding inter­actions between tellurium atoms of the central ring and the pyrazine N atoms of adjacent mol­ecules result in a supra­molecular helix motif.

## Graphical abstract

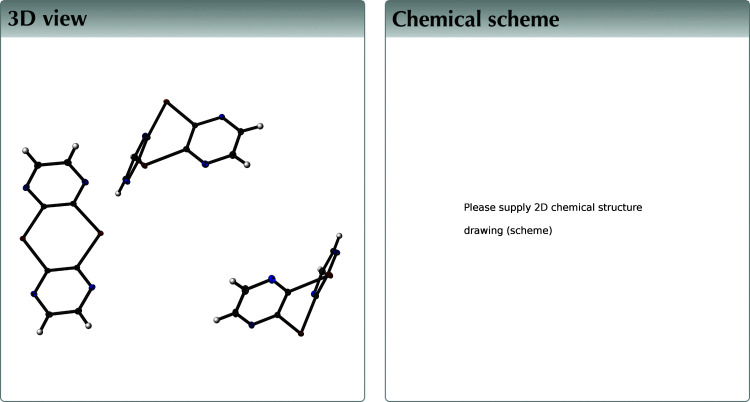

## Structure description

Heterocyclic tellurium compounds have found considerable attention due to their tendency to form supra­molecular assemblies including mol­ecular wires (Kremer *et al.*, 2016 ▸), ribbons (Cozzolino *et al*., 2010 ▸) and rings (Ho *et al.*, 2016 ▸, 2017 ▸). Such assembles can give rise to materials with non-linear optical properties (Cozzolino *et al*., 2010 ▸), as well as novel phospho­rescent organic emitters (Kremer *et al.*, 2015 ▸). A ribbon motif resulting from secondary inter­molecular N⋯Te bonding inter­actions of 2.767 (6) and 2.659 (6) Å was reported for 3,4-di­cyano-1,2,5-tellura­diazole (Cozzolino *et al.*, 2010 ▸). Similarly, mol­ecular wire motifs resulting from secondary inter­molecular N⋯Te bonding were observed for 2-substituted benzo-1,3-tellurazoles, but with significantly longer N⋯Te distances. This is exemplified by 2-(2-furan­yl) benzo-1,3-tellurazole, 3.17 Å (Kremer *et al*., 2016 ▸) and 1,3-benzotellurazol-2-ylaceto­nitrile, 3.16 Å (Sanford *et al*., 2017 ▸). Not all Te, N-containing heterocycles form supra­molecular wires or ribbons. Thus, 10*H*-pyrazino­[2,3-*b*][1,4]benzotellurazine (Smith *et al.*, 2020 ▸), 2*H*-1,4-benzo-tellurazin-3(4*H*)-one and 2,3-di­hydro-1,5-benzotellurazepin-4(5*H*)-one (Myers *et al.*, 2016 ▸) lack this feature. The [1,4]dichalcogena[2,3-*b*:5,6-*b*′]di­pyrazines remain poorly explored and no examples containing heavy chalcogens were reported prior to this study.

**Figure 1:**
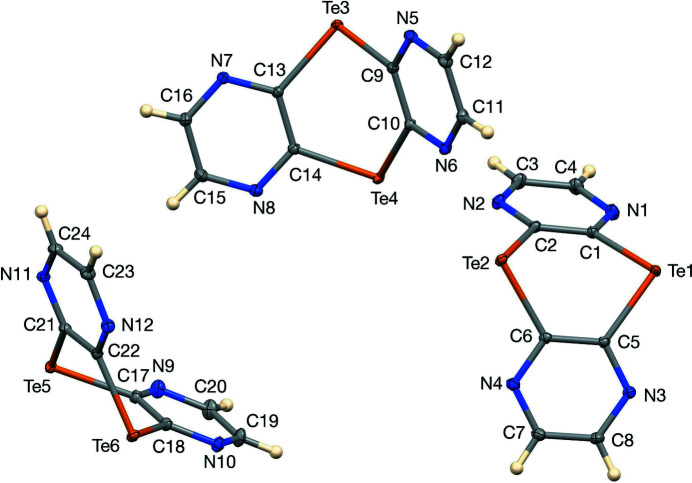
The asymmetric unit of [1,4]ditellurino[2,3-*b*:5,6-*b*′]di­pyrazine with 50% ellipsoids.

The three mol­ecules of the asymmetric unit are shown in Fig. 1 ▸, which illustrates their folded V shapes. The degree of folding along the Te⋯Te line can be described by φ, the dihedral angle between the two C_2_Te_2_ moieties of the central ring. This dihedral angle has a value of 60.08 (5)° for the mol­ecule containing Te1 and Te2, 57.16 (5)° for the Te3/Te4 mol­ecule, and 56.54 (5)° for the Te5/Te6 mol­ecule, with a mean value of 57.9°. A sulfur analog of the title compound has been structurally characterized (Lynch *et al.*, 1994 ▸), but is planar rather than folded along the chalcogen–chalcogen axis. The corresponding selenium congener remains unreported. The shape of the title compound shows structural similarity to those of 9,10-dichalcogenanthracenes containing tellurium and one other chalcogen atom in the central ring (Dereu *et al.*, 1981 ▸; Meyers *et al.*, 1988 ▸), as well as to the recently characterized 10*H*-pyrazino­[2,3-*b*][1,4]benzotellurazine (Smith *et al.*, 2020 ▸). All are V-shaped, but the extent to which the center ring is folded varies considerably. The title compound and telluranthrene (φ = 57.86°) are nearly identical in this respect, while analogous compounds containing nitro­gen as one apex heteroatom show much a less pronounced V shape. This is exemplified by dibenzo[*b*,*e*]tellurazine, with φ = 18.28°(Junk *et al.*, 1993 ▸) and 10*H*-pyrazino­[2,3-b][1,4]benzotellurazine, φ = 18.29° (Smith *et al.*, 2020 ▸) for the central C_2_TeN moieties.

The C—Te—C angles for the three independent mol­ecules of the title compound range from 91.48 (6) to 93.80 (6)°, similar to those of 95.3 and 95.9°, respectively, previously reported for telluranthrene (Dereu *et al.*, 1981 ▸). C—Te bond lengths range from 2.1105 (16) Å to 2.1381 (17) Å, in good agreement with those predicted by their covalent radii.

**Figure 2:**
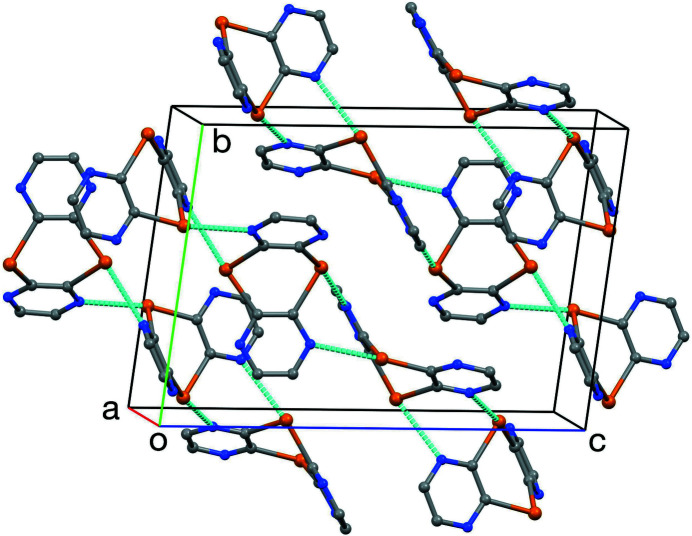
The unit cell, showing inter­molecular Te⋯N contacts.

Inter­molecular features are dominated by Te⋯N inter­actions involving all Te atoms, as shown in Fig. 2 ▸. The range of distances for these contacts is 2.894 (2) to 2.963 (2) Å. These fall between those of 2.767 (6) and 2.659 (6) Å reported for 3,4-di­cyano-1,2,5-tellura­diazole (Cozzolino *et al.*, 2010 ▸) and those for benzo-1,3-tellurazoles, ranging from 2.985 Å for 2-(methyl­sulfan­yl)-1,3-benzotellurazole (Ali *et al.*, 2016 ▸) to 3.169 Å for 2-(2-fur­yl)-1,3-benzotellurazole (Kremer *et al*., 2016 ▸). In contrast, despite its structural similarity, 10*H*-pyrazino­[2,3-*b*][1,4]benzotellurazine does not exhibit any supra­molecular Te⋯N bonding but forms hydrogen-bonded dimers instead (Smith *et al.*, 2020 ▸).

**Figure 3:**
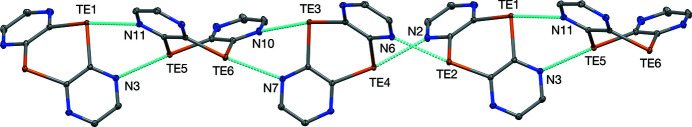
A portion of the helical chain, side view.

Each mol­ecule of the title compound is involved in four Te⋯N contacts, forming helical chains, as shown in Figs. 3 ▸ and 4 ▸. The helices have approximate threefold helical symmetry, with a three-mol­ecule repeat period. The helical chains are in the [1 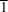 1] direction and have a repeat distance of 20.244 (2) Å.

**Figure 5:**
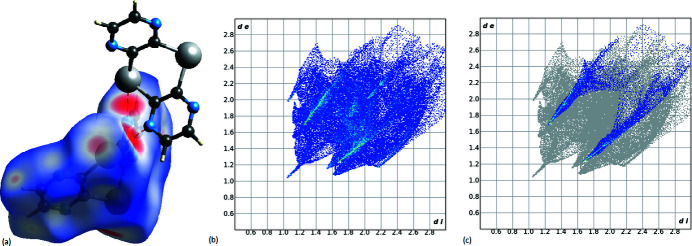
(*a*) Hirshfeld surface mapped over d_norm_, (*b*) two-dimensional fingerprint plot, (*c*) two-dimensional fingerprint plot with reciprocal N· · · Te contacts highlighted.

The Hirshfeld surface enclosing the Te3/Te4 mol­ecule was calculated with respect to *d*_e_, *d*_i_ and *d*_norm_ using *Crystal Explorer* (Spackman *et al.*, 2021 ▸), where *d*_e_ and *d*_i_ represent the nearest distance of external or inter­nal nucleus from a point of inter­est on the iso-surface. The dominant N⋯Te inter­actions with the adjacent Te1/Te2 mol­ecule can be seen as the bright red areas on the Hirshfeld surface. The two-dimensional fingerprint plot and a two-dimensional fingerprint plot highlighting close reciprocal N⋯Te contacts are shown in Fig. 5 ▸. These contacts include 14.6% of the surface area.

A search of the Cambridge Structural Database (May 2021 update; Groom *et al.*, 2016 ▸) for similar organochalcogen heterocycles yielded 9,10-dichalcogenaanthracenes, C_12_H_8_(*X*,*Y*), (*X*,*Y*) = (O, Te), (S, Te), (Se, Te) and (Te, Te): PXTELL (Smith *et al.*, 1973 ▸), VEHVUZ (Meyers *et al.*, 1988 ▸), VEHWEK (Meyers *et al.*, 1988 ▸), and BAVJIR (Dereu *et al.*, 1981 ▸), respectively. A further comparison was carried out with the sulfur analog of the title compound, WIBWEJ (Lynch *et al.*, 1994 ▸), as well as with benzo[1,4]tellurazine derivatives HABJID (Junk *et al.*, 1993 ▸), UGIHIEL (Smith *et al.*, 2020 ▸) and BUTNOV (Myers *et al*., 2016 ▸). A comparison with other Te, N-containing heterocycles known to undergo supra­molecular Te⋯N bonding included 1,3-benzotellurazoles OLUQIX (Kremer *et al.*, 2016 ▸), RUVWUC (Kremer *et al.*, 2015 ▸), HALWID (Sanford *et al.*, 2017 ▸) and 3,4-di­cyano-1,2,5-tellura­diazole AREGEK01 (Semenov *et al.*, 2012 ▸).

## Synthesis and crystallization

**Preparation of [1,4]ditellurino[2,3-** ***b*** **:5,6-** ***b*** **′]di­pyrazine**: a 100 ml round-bottom flask equipped with mechanical stirring and inert gas inlet was charged with tellurium power (200 mesh, 1.28 g, 10 mmol), sodium hydride (0.6 g of 60% emulsion in mineral oil, 15 mmol) and dry *N*-methyl-2-pyrrolidone (12 ml). The mixture was purged with nitro­gen, placed in a Wood’s metal bath and heated to 453 K with mechanical stirring for two hours. 2,3-Di­chloro­pyrazine (1.49 g, 10 mmol) was then added, followed by continued stirring at 453 K. The mixture was allowed to cool and diluted with water (100 ml). Solids were collected by filtration and dried. They were subsequently extracted with 2 × 10 ml of chloro­form. The combined extracts were chromatographed on a 1.5 × 10 cm column (silica gel, neutral, 200 mesh) using chloro­form as mobile phase, followed by chloro­form: aceto­nitrile (10:1 *v*/*v*). A yellow band eluted first and was identified as bis­(pyrazin-2-yl)tellurium by mass spectrometry. This was followed by a blue band, identified as bis­(3-chloro­pyrazin-2-yl)ditellurium. The following yellow band contained the title compound. Crystallization from chloro­form solution furnished yellow crystals, m.p. 413–415 K, yield 46 mg (2.2%).

**Properties:** ^1^H NMR (CDCl_3_, p.p.m.): 8.31 (*s*, 4H). ^13^C NMR (CDCl_3_, p.p.m.): 1443.46, 154.32. The compound slowly oxidizes when exposed to air in solution. A sample suitable for X-ray crystallography was obtained by evaporation of a solution in chloro­form.

## Refinement

Crystal data, data collection and structure refinement details are summarized in Table 1 ▸. In the later stages of refinement, a small amount of twinning was detected, by 180° rotation about the reciprocal 110 direction. Final refinement was as a twin-component twin using an *HKL5* file prepared by *ROTAX* (Parsons *et al.*, 2003 ▸). The BASF parameter is 0.0250 (4).

## Supplementary Material

Crystal structure: contains datablock(s) I. DOI: 10.1107/S2414314622006228/hb4407sup1.cif

Structure factors: contains datablock(s) I. DOI: 10.1107/S2414314622006228/hb4407Isup2.hkl

Supporting information file. DOI: 10.1107/S2414314622006228/hb4407Isup3.cml

CCDC reference: 2179053

Additional supporting information:  crystallographic information; 3D view; checkCIF report

## Figures and Tables

**Figure 4:**
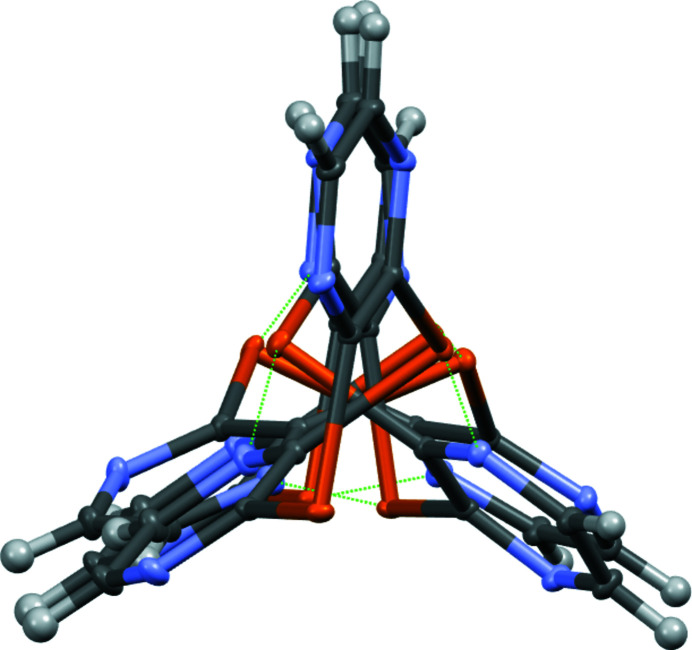
View of chain along the helix axis.

**Table 1:** Experimental details

Crystal data	
Chemical formula	C_8_H_4_N_4_Te_2_
*M*_r_	411.35
Crystal system, space group	Triclinic, *P* 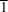
Temperature (K)	90
*a*, *b*, *c* (Å)	7.6531 (8), 11.7862 (12), 16.8371 (18)
α, β, γ (°)	81.350 (2), 85.884 (2), 80.440 (2)
*V* (Å^3^)	1478.9 (3)
*Z*	6
Radiation type	Mo *K*α
μ (mm^−1^)	5.88
Crystal size (mm)	0.19 × 0.17 × 0.16

Data collection	
Diffractometer	Bruker Kappa APEXII DUO CCD
Absorption correction	Multi-scan (*SADABS*; Krause *et al.*, 2015 ▸)
*T*_min_, *T*_max_	0.362, 0.453
No. of measured, independent and observed [*I* > 2σ(*I*)] reflections	158610, 158610, 145750
*R*_int_	0.025
(sin θ/λ)_max_ (Å^−1^)	0.950

Refinement	
*R*[*F* ^2^ > 2σ(*F* ^2^)], *wR*(*F* ^2^), *S*	0.024, 0.058, 1.10
No. of reflections	158610
No. of parameters	381
H-atom treatment	H-atom parameters constrained
Δρ_max_, Δρ_min_ (e Å^−3^)	2.18, −0.97

Computer programs: *APEX3* and *SAINT* (Bruker, 2016 ▸), *SHELXT2014/5* (Sheldrick, 2015*a* ▸), *SHELXL2018/1* (Sheldrick, 2015*b* ▸), *Mercury* (Macrae *et al.*, 2020 ▸), and *publCIF* (Westrip, 2010 ▸).

## full crystallographic data

### Crystal data

**Table:**

C_8_H_4_N_4_Te_2_	*Z* = 6
*M**_r_* = 411.35	*F*(000) = 1104
Triclinic, *P*1	*D*_x_ = 2.771 Mg m^−^^3^
*a* = 7.6531 (8) Å	Mo *K*α radiation, λ = 0.71073 Å
*b* = 11.7862 (12) Å	Cell parameters from 9342 reflections
*c* = 16.8371 (18) Å	θ = 2.8–42.1°
α = 81.350 (2)°	µ = 5.88 mm^−^^1^
β = 85.884 (2)°	*T* = 90 K
γ = 80.440 (2)°	Fragment, yellow
*V* = 1478.9 (3) Å^3^	0.19 × 0.17 × 0.16 mm

### Data collection

**Table:**

Bruker Kappa APEXII DUO CCD diffractometer	158610 independent reflections
Radiation source: fine-focus sealed tube	145750 reflections with *I* > 2σ(*I*)
TRIUMPH curved graphite monochromator	*R*_int_ = 0.025
φ and ω scans	θ_max_ = 42.5°, θ_min_ = 1.2°
Absorption correction: multi-scan (SADABS; Krause *et al.*, 2015)	*h* = −14→14
*T*_min_ = 0.362, *T*_max_ = 0.453	*k* = −22→22
158610 measured reflections	*l* = −31→31

### Refinement

**Table:**

Refinement on *F*^2^	Secondary atom site location: difference Fourier map
Least-squares matrix: full	Hydrogen site location: inferred from neighbouring sites
*R*[*F*^2^ > 2σ(*F*^2^)] = 0.024	H-atom parameters constrained
*wR*(*F*^2^) = 0.058	*w* = 1/[σ^2^(*F*_o_^2^) + (0.0009*P*)^2^ + 1.6438*P*] where *P* = (*F*_o_^2^ + 2*F*_c_^2^)/3
*S* = 1.10	(Δ/σ)_max_ = 0.002
158610 reflections	Δρ_max_ = 2.18 e Å^−^^3^
381 parameters	Δρ_min_ = −0.97 e Å^−^^3^
0 restraints	Extinction correction: SHELXL-2018/1 (Sheldrick 2015b), Fc^*^=kFc[1+0.001xFc^2^λ^3^/sin(2θ)]^-1/4^
Primary atom site location: structure-invariant direct methods	Extinction coefficient: 0.00036 (7)

### Special details

**Table:**

Geometry. All esds (except the esd in the dihedral angle between two l.s. planes) are estimated using the full covariance matrix. The cell esds are taken into account individually in the estimation of esds in distances, angles and torsion angles; correlations between esds in cell parameters are only used when they are defined by crystal symmetry. An approximate (isotropic) treatment of cell esds is used for estimating esds involving l.s. planes.
Refinement. Refined as a two-component twin using an HKL5 file prepared by ROTAX (Parsons *et al.*, 2003). The BASF parameter is 0.0250 (4). All H atoms were located in difference maps and then treated as riding in geometrically idealized positions with C—H distances 0.95 Å and with *U*_iso_(H) =1.2*U*_eq_ for the attached C atom.

### Fractional atomic coordinates and isotropic or equivalent isotropic displacement parameters (Å^2^)

**Table:**

	*x*	*y*	*z*	*U*_iso_*/*U*_eq_	
Te1	0.49841 (2)	0.50998 (2)	0.85722 (2)	0.01013 (2)	
Te2	0.50106 (2)	0.49957 (2)	0.63430 (2)	0.00816 (2)	
N1	0.4741 (2)	0.75856 (14)	0.79682 (9)	0.0131 (3)	
N2	0.4700 (2)	0.75553 (14)	0.63202 (9)	0.0113 (2)	
N3	0.8440 (2)	0.35839 (14)	0.83665 (9)	0.0103 (2)	
N4	0.8595 (2)	0.37038 (14)	0.66970 (9)	0.0104 (2)	
C1	0.4830 (2)	0.65832 (15)	0.76698 (10)	0.0098 (3)	
C2	0.4806 (2)	0.65701 (15)	0.68373 (10)	0.0090 (2)	
C3	0.4596 (3)	0.85524 (16)	0.66225 (11)	0.0131 (3)	
H3	0.450785	0.926470	0.626760	0.016*	
C4	0.4615 (3)	0.85661 (17)	0.74470 (11)	0.0139 (3)	
H4	0.453565	0.928841	0.764348	0.017*	
C5	0.7087 (2)	0.41601 (15)	0.79341 (10)	0.0086 (2)	
C6	0.7142 (2)	0.41927 (15)	0.70924 (10)	0.0084 (2)	
C7	0.9955 (2)	0.31457 (16)	0.71338 (11)	0.0118 (3)	
H7	1.100495	0.280085	0.687036	0.014*	
C8	0.9850 (2)	0.30641 (17)	0.79687 (11)	0.0118 (3)	
H8	1.080803	0.262596	0.826477	0.014*	
Te3	0.02141 (2)	0.96211 (2)	0.37609 (2)	0.00927 (2)	
Te4	0.45479 (2)	0.80313 (2)	0.45840 (2)	0.00845 (2)	
N5	−0.1035 (2)	0.76067 (14)	0.47346 (9)	0.0109 (2)	
N6	0.2130 (2)	0.64559 (13)	0.53966 (9)	0.0094 (2)	
N7	0.2250 (2)	0.92573 (13)	0.22221 (8)	0.0094 (2)	
N8	0.5463 (2)	0.81350 (15)	0.28562 (9)	0.0116 (2)	
C9	0.0482 (2)	0.80356 (15)	0.45639 (10)	0.0087 (2)	
C10	0.2078 (2)	0.74554 (15)	0.48943 (9)	0.0084 (2)	
C11	0.0613 (2)	0.60249 (16)	0.55555 (10)	0.0111 (3)	
H11	0.061189	0.531090	0.590301	0.013*	
C12	−0.0961 (2)	0.65951 (17)	0.52251 (11)	0.0120 (3)	
H12	−0.201159	0.625984	0.534990	0.014*	
C13	0.2442 (2)	0.90429 (15)	0.30184 (10)	0.0085 (2)	
C14	0.4045 (2)	0.84565 (15)	0.33377 (10)	0.0088 (2)	
C15	0.5256 (2)	0.83823 (18)	0.20617 (11)	0.0134 (3)	
H15	0.623674	0.818218	0.170301	0.016*	
C16	0.3656 (2)	0.89205 (16)	0.17491 (10)	0.0111 (3)	
H16	0.355508	0.905375	0.118238	0.013*	
Te5	0.92057 (2)	0.34134 (2)	0.00428 (2)	0.00896 (2)	
Te6	0.93205 (2)	0.03850 (2)	0.12002 (2)	0.00860 (2)	
N9	0.9987 (2)	0.38464 (15)	0.16384 (9)	0.0133 (3)	
N10	1.0234 (2)	0.16105 (14)	0.24945 (9)	0.0118 (2)	
N11	0.5643 (2)	0.29991 (14)	−0.02659 (9)	0.0100 (2)	
N12	0.5688 (2)	0.08150 (14)	0.06426 (9)	0.0105 (2)	
C17	0.9704 (2)	0.29510 (15)	0.12895 (10)	0.0094 (3)	
C18	0.9813 (2)	0.18233 (15)	0.17227 (10)	0.0092 (2)	
C19	1.0551 (3)	0.25069 (17)	0.28327 (11)	0.0143 (3)	
H19	1.087619	0.237944	0.337674	0.017*	
C20	1.0417 (3)	0.36185 (18)	0.24084 (11)	0.0155 (3)	
H20	1.063593	0.423560	0.267200	0.019*	
C21	0.7081 (2)	0.24611 (15)	0.01189 (10)	0.0084 (2)	
C22	0.7113 (2)	0.13526 (15)	0.05717 (10)	0.0085 (2)	
C23	0.4234 (2)	0.13848 (17)	0.02780 (11)	0.0117 (3)	
H23	0.318870	0.103801	0.033307	0.014*	
C24	0.4216 (2)	0.24684 (17)	−0.01780 (11)	0.0115 (3)	
H24	0.316408	0.284193	−0.043376	0.014*	

### Atomic displacement parameters (Å^2^)

**Table:**

	*U*^11^	*U*^22^	*U*^33^	*U*^12^	*U*^13^	*U*^23^
Te1	0.01103 (4)	0.00946 (4)	0.00761 (4)	0.00239 (3)	0.00198 (3)	0.00057 (3)
Te2	0.00895 (4)	0.00699 (4)	0.00847 (4)	−0.00041 (3)	−0.00201 (3)	−0.00098 (3)
N1	0.0182 (7)	0.0093 (6)	0.0112 (6)	−0.0003 (5)	0.0013 (5)	−0.0026 (5)
N2	0.0145 (6)	0.0085 (6)	0.0105 (5)	−0.0016 (5)	−0.0021 (5)	0.0004 (4)
N3	0.0089 (5)	0.0111 (6)	0.0099 (5)	0.0000 (5)	−0.0007 (4)	−0.0002 (5)
N4	0.0096 (6)	0.0105 (6)	0.0112 (5)	−0.0001 (5)	−0.0001 (4)	−0.0036 (5)
C1	0.0107 (6)	0.0087 (6)	0.0089 (6)	0.0004 (5)	0.0004 (5)	−0.0005 (5)
C2	0.0096 (6)	0.0075 (6)	0.0094 (6)	−0.0007 (5)	−0.0006 (5)	0.0000 (5)
C3	0.0173 (8)	0.0082 (7)	0.0133 (7)	−0.0022 (6)	−0.0014 (6)	0.0003 (5)
C4	0.0188 (8)	0.0092 (7)	0.0136 (7)	−0.0013 (6)	0.0008 (6)	−0.0026 (5)
C5	0.0081 (6)	0.0078 (6)	0.0093 (6)	−0.0006 (5)	0.0001 (5)	−0.0002 (5)
C6	0.0086 (6)	0.0071 (6)	0.0094 (6)	−0.0008 (5)	−0.0010 (5)	−0.0014 (5)
C7	0.0100 (6)	0.0122 (7)	0.0130 (6)	0.0000 (5)	−0.0003 (5)	−0.0034 (5)
C8	0.0090 (6)	0.0128 (7)	0.0126 (6)	0.0013 (5)	−0.0012 (5)	−0.0009 (5)
Te3	0.00845 (4)	0.00910 (4)	0.00846 (4)	0.00204 (3)	0.00024 (3)	0.00044 (3)
Te4	0.00689 (4)	0.00985 (4)	0.00835 (4)	−0.00251 (3)	−0.00212 (3)	0.00166 (3)
N5	0.0080 (5)	0.0141 (6)	0.0107 (5)	−0.0020 (5)	0.0006 (4)	−0.0018 (5)
N6	0.0103 (6)	0.0087 (6)	0.0089 (5)	−0.0019 (5)	−0.0009 (4)	0.0003 (4)
N7	0.0099 (6)	0.0096 (6)	0.0083 (5)	−0.0003 (5)	−0.0007 (4)	−0.0009 (4)
N8	0.0082 (6)	0.0145 (7)	0.0113 (6)	−0.0006 (5)	0.0000 (4)	−0.0011 (5)
C9	0.0078 (6)	0.0099 (6)	0.0080 (5)	−0.0007 (5)	−0.0003 (4)	−0.0008 (5)
C10	0.0081 (6)	0.0089 (6)	0.0081 (5)	−0.0019 (5)	−0.0005 (5)	−0.0004 (5)
C11	0.0126 (7)	0.0110 (7)	0.0099 (6)	−0.0042 (6)	0.0010 (5)	0.0001 (5)
C12	0.0099 (6)	0.0139 (7)	0.0127 (6)	−0.0042 (6)	0.0006 (5)	−0.0015 (6)
C13	0.0088 (6)	0.0082 (6)	0.0082 (5)	−0.0008 (5)	−0.0001 (5)	−0.0006 (5)
C14	0.0077 (6)	0.0089 (6)	0.0094 (6)	−0.0018 (5)	−0.0007 (5)	0.0001 (5)
C15	0.0097 (7)	0.0182 (8)	0.0112 (6)	0.0002 (6)	0.0017 (5)	−0.0019 (6)
C16	0.0109 (7)	0.0130 (7)	0.0090 (6)	−0.0010 (5)	−0.0001 (5)	−0.0011 (5)
Te5	0.00776 (4)	0.01048 (5)	0.00853 (4)	−0.00312 (3)	−0.00108 (3)	0.00122 (3)
Te6	0.00945 (4)	0.00712 (4)	0.00926 (4)	−0.00022 (3)	−0.00264 (3)	−0.00145 (3)
N9	0.0172 (7)	0.0121 (7)	0.0120 (6)	−0.0066 (5)	−0.0009 (5)	−0.0015 (5)
N10	0.0157 (7)	0.0108 (6)	0.0091 (5)	−0.0012 (5)	−0.0026 (5)	−0.0022 (5)
N11	0.0072 (5)	0.0115 (6)	0.0106 (5)	0.0000 (5)	−0.0007 (4)	−0.0004 (5)
N12	0.0103 (6)	0.0116 (6)	0.0103 (5)	−0.0034 (5)	−0.0002 (4)	−0.0023 (5)
C17	0.0091 (6)	0.0108 (7)	0.0086 (6)	−0.0028 (5)	−0.0009 (5)	−0.0008 (5)
C18	0.0097 (6)	0.0090 (6)	0.0091 (6)	−0.0013 (5)	−0.0011 (5)	−0.0019 (5)
C19	0.0207 (8)	0.0139 (8)	0.0096 (6)	−0.0043 (6)	−0.0030 (6)	−0.0028 (6)
C20	0.0216 (9)	0.0142 (8)	0.0133 (7)	−0.0083 (7)	−0.0019 (6)	−0.0040 (6)
C21	0.0067 (6)	0.0098 (6)	0.0086 (6)	−0.0010 (5)	−0.0006 (4)	−0.0010 (5)
C22	0.0086 (6)	0.0087 (6)	0.0082 (6)	−0.0009 (5)	−0.0011 (5)	−0.0020 (5)
C23	0.0090 (6)	0.0140 (7)	0.0130 (6)	−0.0036 (5)	−0.0003 (5)	−0.0029 (6)
C24	0.0072 (6)	0.0144 (7)	0.0124 (6)	−0.0005 (5)	−0.0011 (5)	−0.0016 (5)

### Geometric parameters (Å, º)

**Table:**

Te1—C5	2.1185 (17)	N8—C15	1.341 (2)
Te1—C1	2.1301 (17)	N8—C14	1.345 (2)
Te2—C2	2.1237 (17)	C9—C10	1.405 (2)
Te2—C6	2.1381 (17)	C11—C12	1.388 (3)
N1—C4	1.336 (3)	C11—H11	0.9500
N1—C1	1.342 (2)	C12—H12	0.9500
N2—C2	1.337 (2)	C13—C14	1.405 (2)
N2—C3	1.338 (2)	C15—C16	1.383 (3)
N3—C8	1.337 (2)	C15—H15	0.9500
N3—C5	1.338 (2)	C16—H16	0.9500
N4—C7	1.339 (2)	Te5—C21	2.1105 (16)
N4—C6	1.345 (2)	Te5—C17	2.1332 (16)
C1—C2	1.406 (2)	Te6—C18	2.1209 (17)
C3—C4	1.392 (3)	Te6—C22	2.1281 (17)
C3—H3	0.9500	N9—C17	1.337 (2)
C4—H4	0.9500	N9—C20	1.337 (3)
C5—C6	1.410 (2)	N10—C19	1.336 (2)
C7—C8	1.392 (3)	N10—C18	1.338 (2)
C7—H7	0.9500	N11—C21	1.334 (2)
C8—H8	0.9500	N11—C24	1.335 (2)
Te3—C13	2.1211 (17)	N12—C23	1.338 (2)
Te3—C9	2.1248 (17)	N12—C22	1.339 (2)
Te4—C10	2.1180 (16)	C17—C18	1.409 (2)
Te4—C14	2.1302 (16)	C19—C20	1.386 (3)
N5—C9	1.338 (2)	C19—H19	0.9500
N5—C12	1.340 (2)	C20—H20	0.9500
N6—C11	1.337 (2)	C21—C22	1.408 (2)
N6—C10	1.339 (2)	C23—C24	1.386 (3)
N7—C16	1.334 (2)	C23—H23	0.9500
N7—C13	1.340 (2)	C24—H24	0.9500
			
C5—Te1—C1	92.54 (6)	N5—C12—C11	121.60 (16)
C2—Te2—C6	91.48 (6)	N5—C12—H12	119.2
C4—N1—C1	117.64 (16)	C11—C12—H12	119.2
C2—N2—C3	117.78 (15)	N7—C13—C14	121.05 (15)
C8—N3—C5	117.52 (15)	N7—C13—Te3	116.77 (12)
C7—N4—C6	117.69 (15)	C14—C13—Te3	122.17 (12)
N1—C1—C2	120.90 (16)	N8—C14—C13	121.25 (15)
N1—C1—Te1	113.35 (12)	N8—C14—Te4	113.13 (12)
C2—C1—Te1	125.74 (13)	C13—C14—Te4	125.59 (12)
N2—C2—C1	120.98 (16)	N8—C15—C16	121.90 (16)
N2—C2—Te2	117.13 (12)	N8—C15—H15	119.0
C1—C2—Te2	121.87 (12)	C16—C15—H15	119.0
N2—C3—C4	121.26 (17)	N7—C16—C15	121.76 (16)
N2—C3—H3	119.4	N7—C16—H16	119.1
C4—C3—H3	119.4	C15—C16—H16	119.1
N1—C4—C3	121.43 (17)	C21—Te5—C17	93.44 (6)
N1—C4—H4	119.3	C18—Te6—C22	93.72 (6)
C3—C4—H4	119.3	C17—N9—C20	117.07 (16)
N3—C5—C6	120.83 (15)	C19—N10—C18	117.32 (16)
N3—C5—Te1	116.33 (12)	C21—N11—C24	117.52 (16)
C6—C5—Te1	122.70 (12)	C23—N12—C22	117.19 (16)
N4—C6—C5	120.95 (15)	N9—C17—C18	121.22 (15)
N4—C6—Te2	114.60 (12)	N9—C17—Te5	113.16 (12)
C5—C6—Te2	124.45 (12)	C18—C17—Te5	125.57 (12)
N4—C7—C8	120.88 (16)	N10—C18—C17	120.98 (15)
N4—C7—H7	119.6	N10—C18—Te6	116.55 (12)
C8—C7—H7	119.6	C17—C18—Te6	122.48 (12)
N3—C8—C7	121.98 (17)	N10—C19—C20	121.55 (17)
N3—C8—H8	119.0	N10—C19—H19	119.2
C7—C8—H8	119.0	C20—C19—H19	119.2
C13—Te3—C9	93.30 (6)	N9—C20—C19	121.84 (17)
C10—Te4—C14	93.80 (6)	N9—C20—H20	119.1
C9—N5—C12	117.13 (16)	C19—C20—H20	119.1
C11—N6—C10	117.26 (15)	N11—C21—C22	121.09 (15)
C16—N7—C13	117.26 (15)	N11—C21—Te5	115.58 (12)
C15—N8—C14	116.70 (16)	C22—C21—Te5	123.27 (12)
N5—C9—C10	121.28 (16)	N12—C22—C21	120.99 (15)
N5—C9—Te3	113.77 (12)	N12—C22—Te6	113.90 (12)
C10—C9—Te3	124.94 (12)	C21—C22—Te6	125.11 (12)
N6—C10—C9	121.09 (15)	N12—C23—C24	121.65 (16)
N6—C10—Te4	115.90 (12)	N12—C23—H23	119.2
C9—C10—Te4	122.91 (12)	C24—C23—H23	119.2
N6—C11—C12	121.62 (16)	N11—C24—C23	121.50 (16)
N6—C11—H11	119.2	N11—C24—H24	119.3
C12—C11—H11	119.2	C23—C24—H24	119.3
			
C4—N1—C1—C2	0.5 (3)	C16—N7—C13—C14	−1.8 (2)
C4—N1—C1—Te1	−179.04 (14)	C16—N7—C13—Te3	178.54 (13)
C3—N2—C2—C1	−0.8 (3)	C15—N8—C14—C13	−1.2 (3)
C3—N2—C2—Te2	−179.06 (13)	C15—N8—C14—Te4	−179.44 (14)
N1—C1—C2—N2	0.2 (3)	N7—C13—C14—N8	2.9 (3)
Te1—C1—C2—N2	179.73 (13)	Te3—C13—C14—N8	−177.51 (13)
N1—C1—C2—Te2	178.42 (13)	N7—C13—C14—Te4	−179.14 (12)
Te1—C1—C2—Te2	−2.1 (2)	Te3—C13—C14—Te4	0.5 (2)
C2—N2—C3—C4	0.6 (3)	C14—N8—C15—C16	−1.3 (3)
C1—N1—C4—C3	−0.7 (3)	C13—N7—C16—C15	−0.7 (3)
N2—C3—C4—N1	0.2 (3)	N8—C15—C16—N7	2.3 (3)
C8—N3—C5—C6	1.3 (2)	C20—N9—C17—C18	1.5 (3)
C8—N3—C5—Te1	−174.55 (13)	C20—N9—C17—Te5	−176.06 (14)
C7—N4—C6—C5	2.7 (2)	C19—N10—C18—C17	−0.2 (3)
C7—N4—C6—Te2	−177.44 (13)	C19—N10—C18—Te6	179.91 (14)
N3—C5—C6—N4	−3.9 (3)	N9—C17—C18—N10	−1.2 (3)
Te1—C5—C6—N4	171.65 (12)	Te5—C17—C18—N10	176.06 (13)
N3—C5—C6—Te2	176.25 (12)	N9—C17—C18—Te6	178.68 (13)
Te1—C5—C6—Te2	−8.2 (2)	Te5—C17—C18—Te6	−4.1 (2)
C6—N4—C7—C8	0.8 (3)	C18—N10—C19—C20	1.2 (3)
C5—N3—C8—C7	2.3 (3)	C17—N9—C20—C19	−0.5 (3)
N4—C7—C8—N3	−3.5 (3)	N10—C19—C20—N9	−0.9 (3)
C12—N5—C9—C10	−0.8 (2)	C24—N11—C21—C22	2.7 (2)
C12—N5—C9—Te3	178.70 (13)	C24—N11—C21—Te5	−174.54 (13)
C11—N6—C10—C9	1.4 (2)	C23—N12—C22—C21	−1.2 (2)
C11—N6—C10—Te4	−175.06 (12)	C23—N12—C22—Te6	178.49 (12)
N5—C9—C10—N6	−0.5 (3)	N11—C21—C22—N12	−1.3 (2)
Te3—C9—C10—N6	−179.93 (12)	Te5—C21—C22—N12	175.69 (12)
N5—C9—C10—Te4	175.68 (12)	N11—C21—C22—Te6	179.06 (12)
Te3—C9—C10—Te4	−3.8 (2)	Te5—C21—C22—Te6	−3.9 (2)
C10—N6—C11—C12	−1.0 (3)	C22—N12—C23—C24	2.2 (3)
C9—N5—C12—C11	1.2 (3)	C21—N11—C24—C23	−1.7 (3)
N6—C11—C12—N5	−0.3 (3)	N12—C23—C24—N11	−0.8 (3)
